# Supplementary figures and images for: Hypermetabolism in mice carrying a near-complete human chromosome 21
Source: eLife. 2023 May 30;12:e86023. doi: 10.7554/eLife.86023 (PMC10229126; doi:10.7554/eLife.86023)

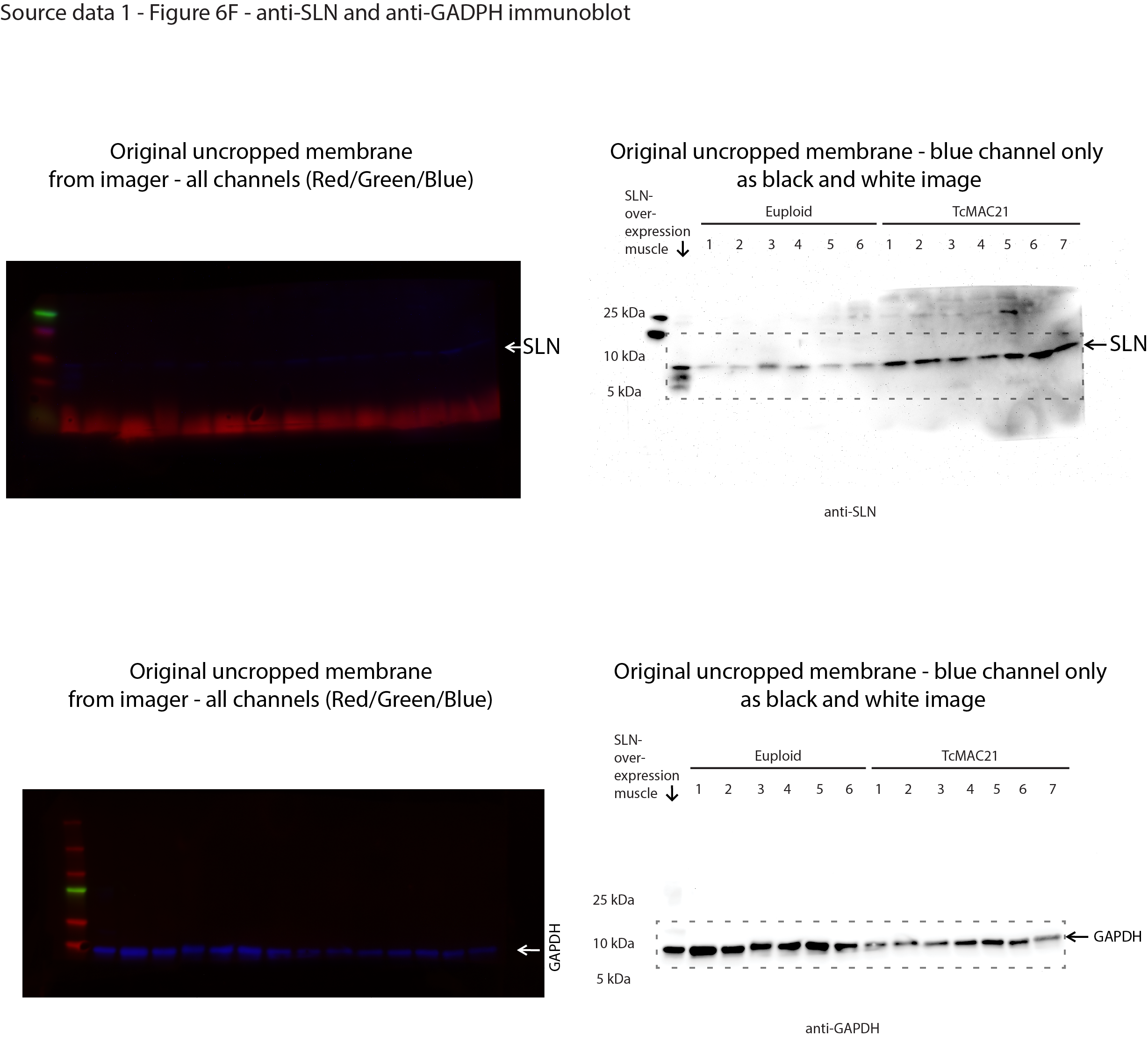

Supplement: Figure 6—source data 1. — Top right—Original uncropped membrane showing a black and white image of the blue channel only. SLN band is marked with an arrow. The dotted outline represents where the membrane image was cropped for use in Figure 6. Bottom left—Original uncropped membrane from imager showing all channels (red/green/blue), with the GAPDH protein band labeled and appearing blue. Bottom right—Original uncropped membrane showing a black and white image of the blue channel only. GAPDH band is marked with an arrow. The dotted outline represents where the membrane image was cropped for use in Figure 6. [file elife-86023-fig6-data1.zip › Figure 6-source data 1/Figure 6-source data 1.tif]

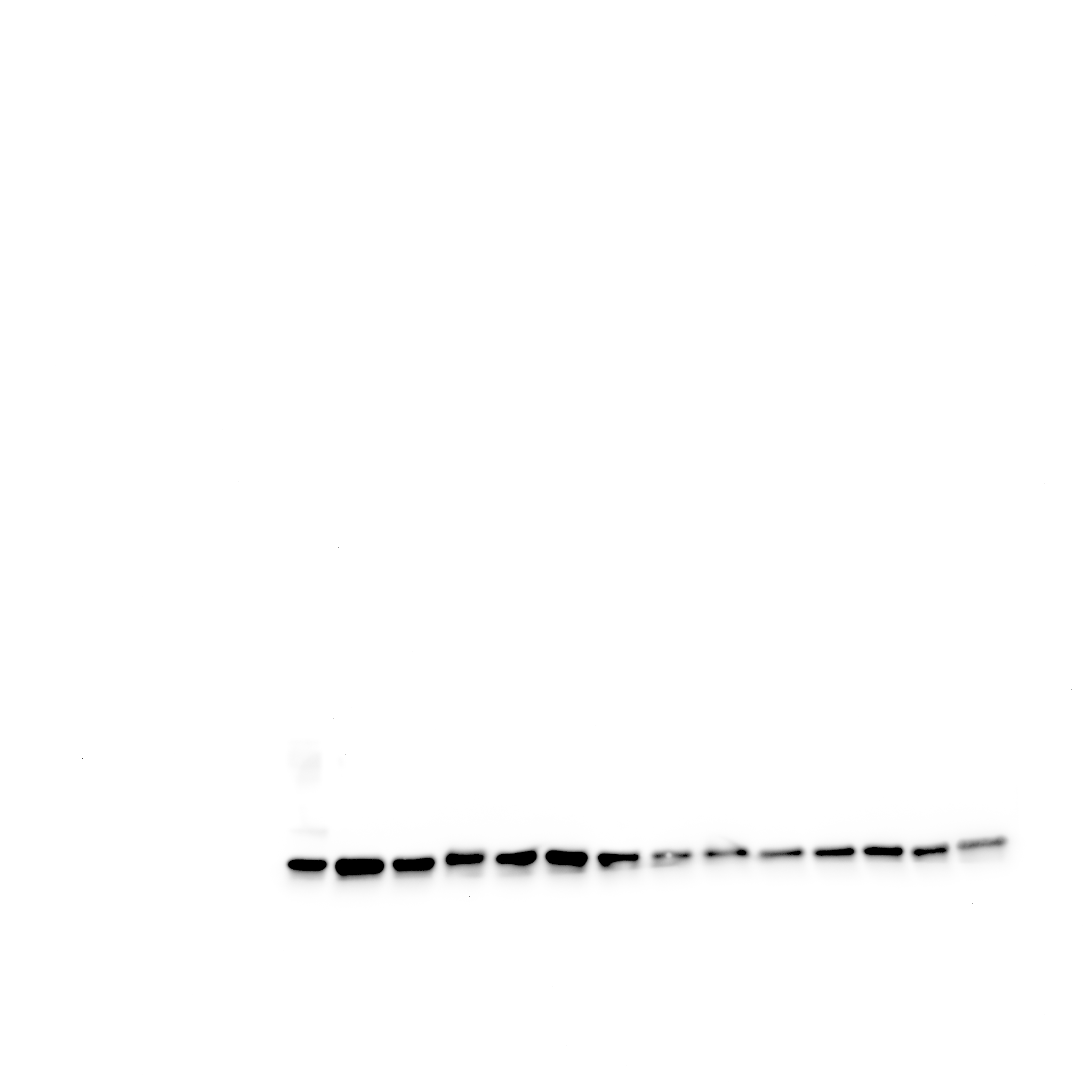

Supplement: Figure 6—source data 1. — Top right—Original uncropped membrane showing a black and white image of the blue channel only. SLN band is marked with an arrow. The dotted outline represents where the membrane image was cropped for use in Figure 6. Bottom left—Original uncropped membrane from imager showing all channels (red/green/blue), with the GAPDH protein band labeled and appearing blue. Bottom right—Original uncropped membrane showing a black and white image of the blue channel only. GAPDH band is marked with an arrow. The dotted outline represents where the membrane image was cropped for use in Figure 6. [file elife-86023-fig6-data1.zip › Figure 6-source data 1/Figure 6-source data 1-Original uncropped membrane - blue channel only as black and white image-antiGAPDH.tif]

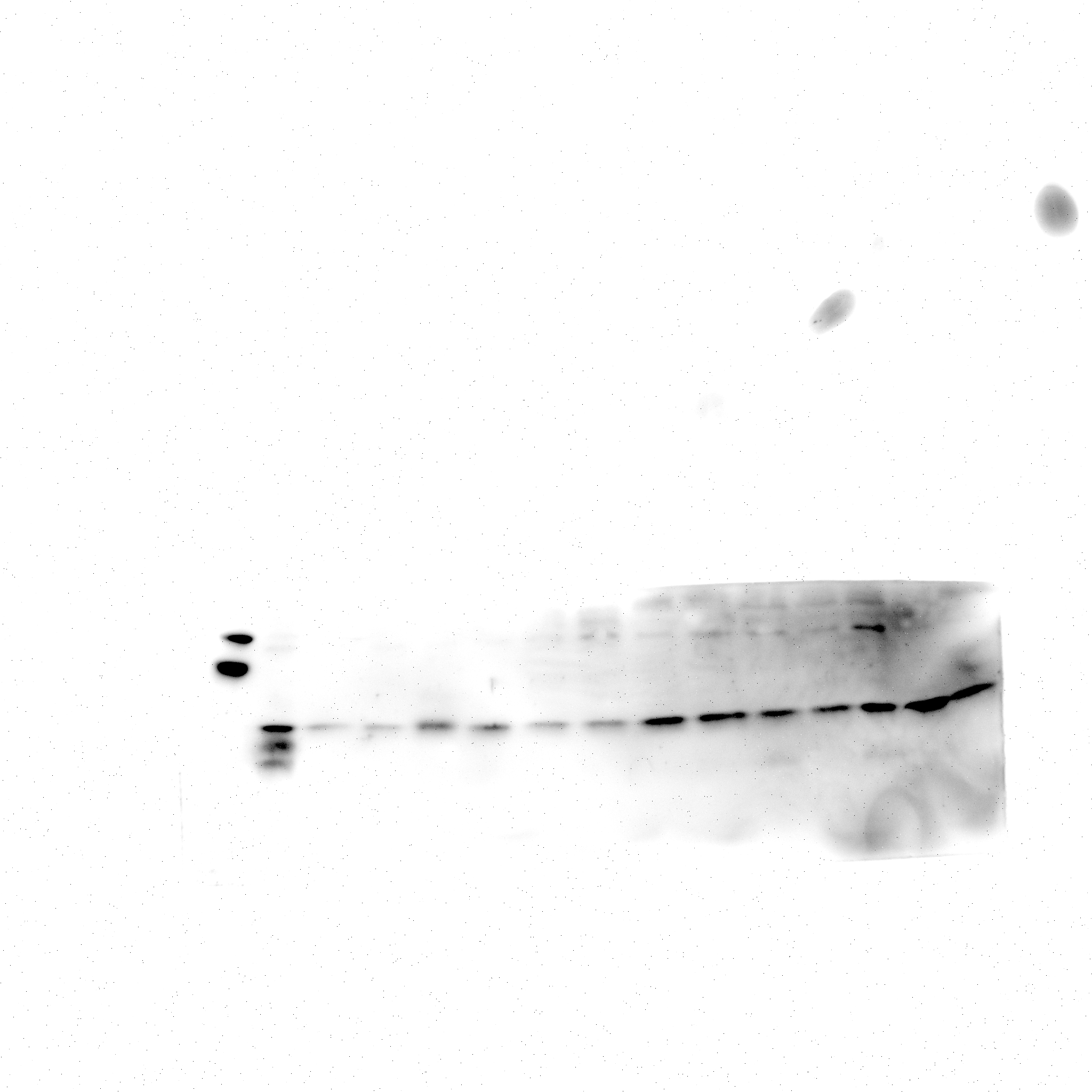

Supplement: Figure 6—source data 1. — Top right—Original uncropped membrane showing a black and white image of the blue channel only. SLN band is marked with an arrow. The dotted outline represents where the membrane image was cropped for use in Figure 6. Bottom left—Original uncropped membrane from imager showing all channels (red/green/blue), with the GAPDH protein band labeled and appearing blue. Bottom right—Original uncropped membrane showing a black and white image of the blue channel only. GAPDH band is marked with an arrow. The dotted outline represents where the membrane image was cropped for use in Figure 6. [file elife-86023-fig6-data1.zip › Figure 6-source data 1/Figure 6-source data 1-Original uncropped membrane - blue channel only as black and white image-antiSLN.tif]

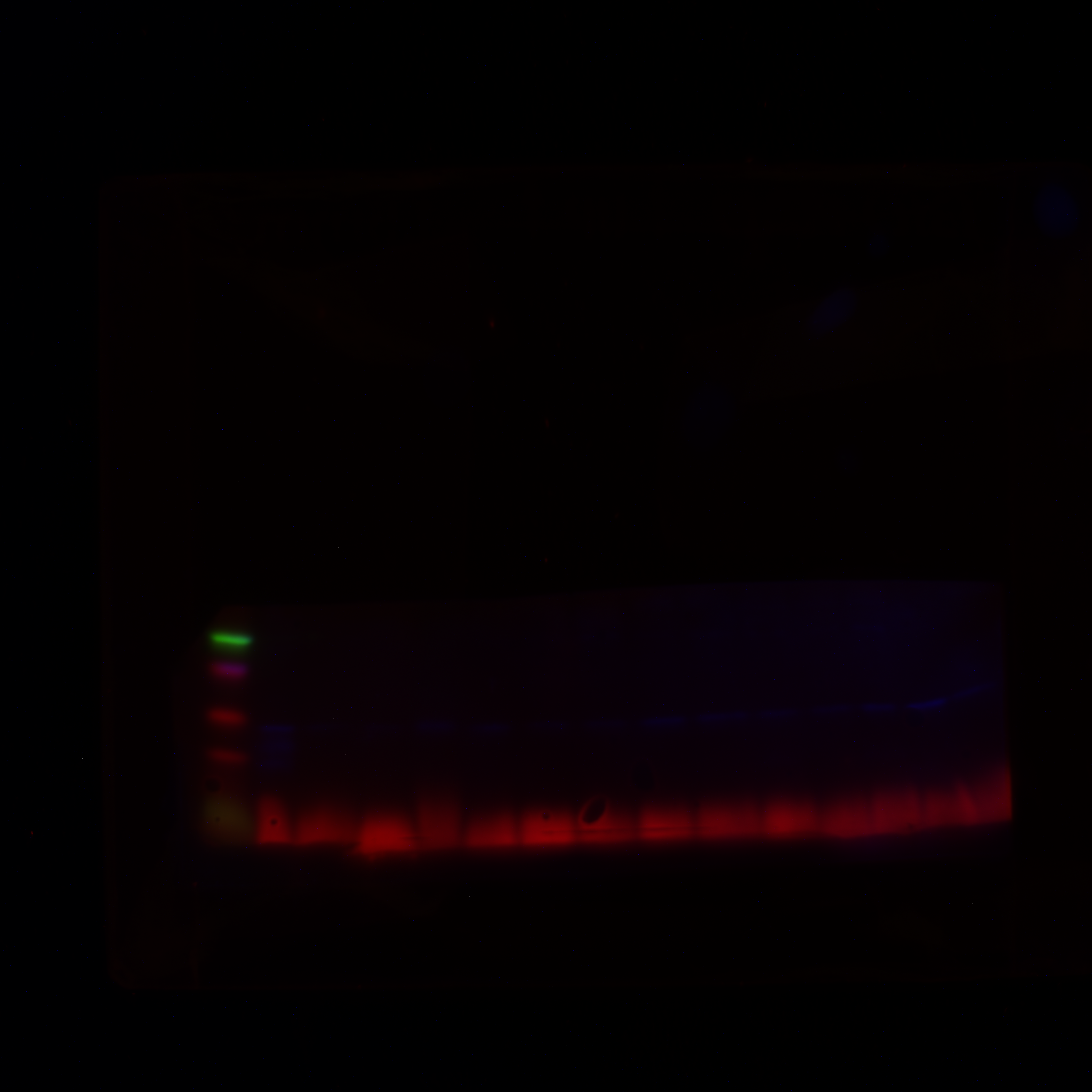

Supplement: Figure 6—source data 1. — Top right—Original uncropped membrane showing a black and white image of the blue channel only. SLN band is marked with an arrow. The dotted outline represents where the membrane image was cropped for use in Figure 6. Bottom left—Original uncropped membrane from imager showing all channels (red/green/blue), with the GAPDH protein band labeled and appearing blue. Bottom right—Original uncropped membrane showing a black and white image of the blue channel only. GAPDH band is marked with an arrow. The dotted outline represents where the membrane image was cropped for use in Figure 6. [file elife-86023-fig6-data1.zip › Figure 6-source data 1/Figure 6-source data 1-Original uncropped membrane from imager - all channels (Red:Green:Blue)-antiSLN.tif]

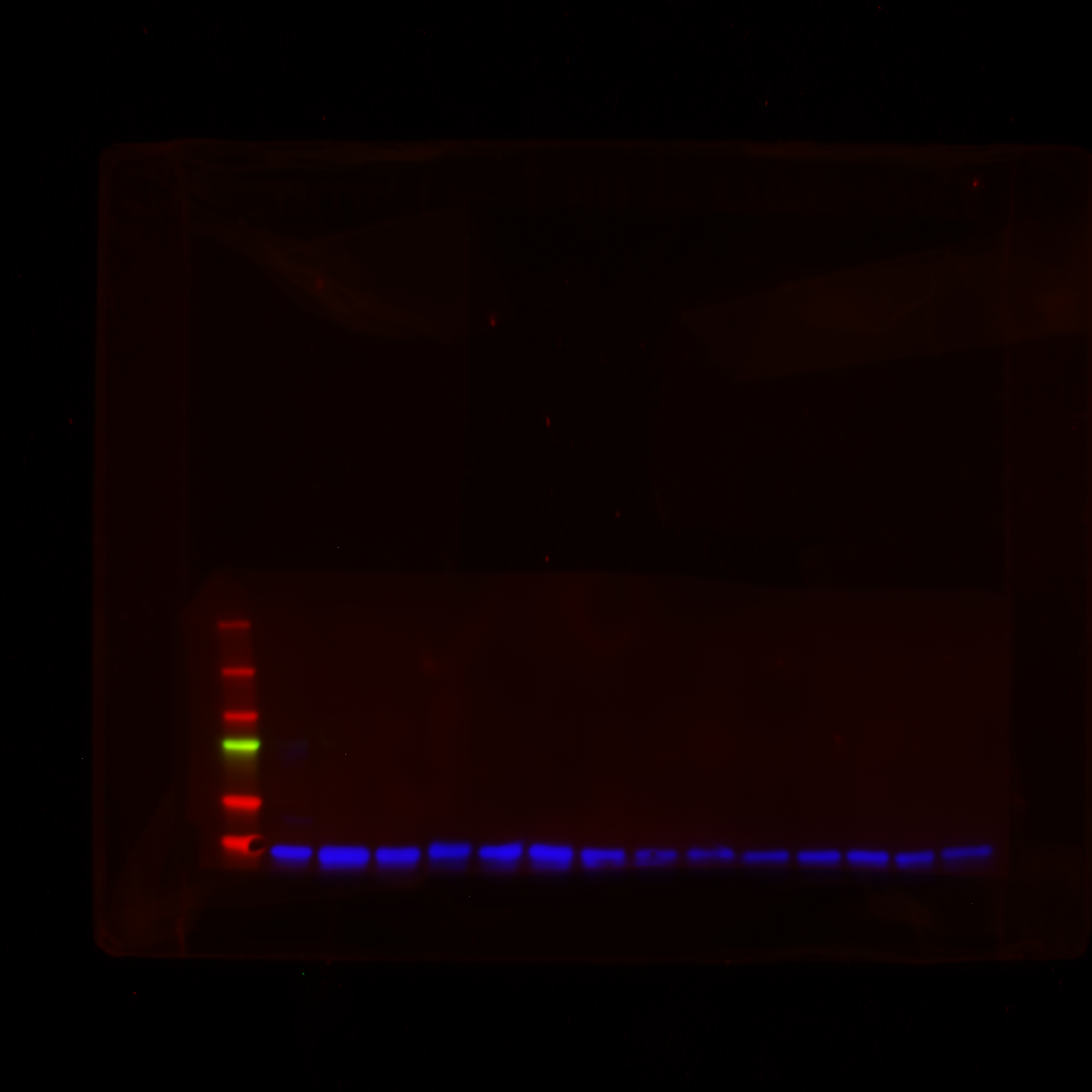

Supplement: Figure 6—source data 1. — Top right—Original uncropped membrane showing a black and white image of the blue channel only. SLN band is marked with an arrow. The dotted outline represents where the membrane image was cropped for use in Figure 6. Bottom left—Original uncropped membrane from imager showing all channels (red/green/blue), with the GAPDH protein band labeled and appearing blue. Bottom right—Original uncropped membrane showing a black and white image of the blue channel only. GAPDH band is marked with an arrow. The dotted outline represents where the membrane image was cropped for use in Figure 6. [file elife-86023-fig6-data1.zip › Figure 6-source data 1/Figure 6-source data 1-Original uncropped membrane from imager - all channels (Red:Green:Blue)-antiGAPDH.tif]

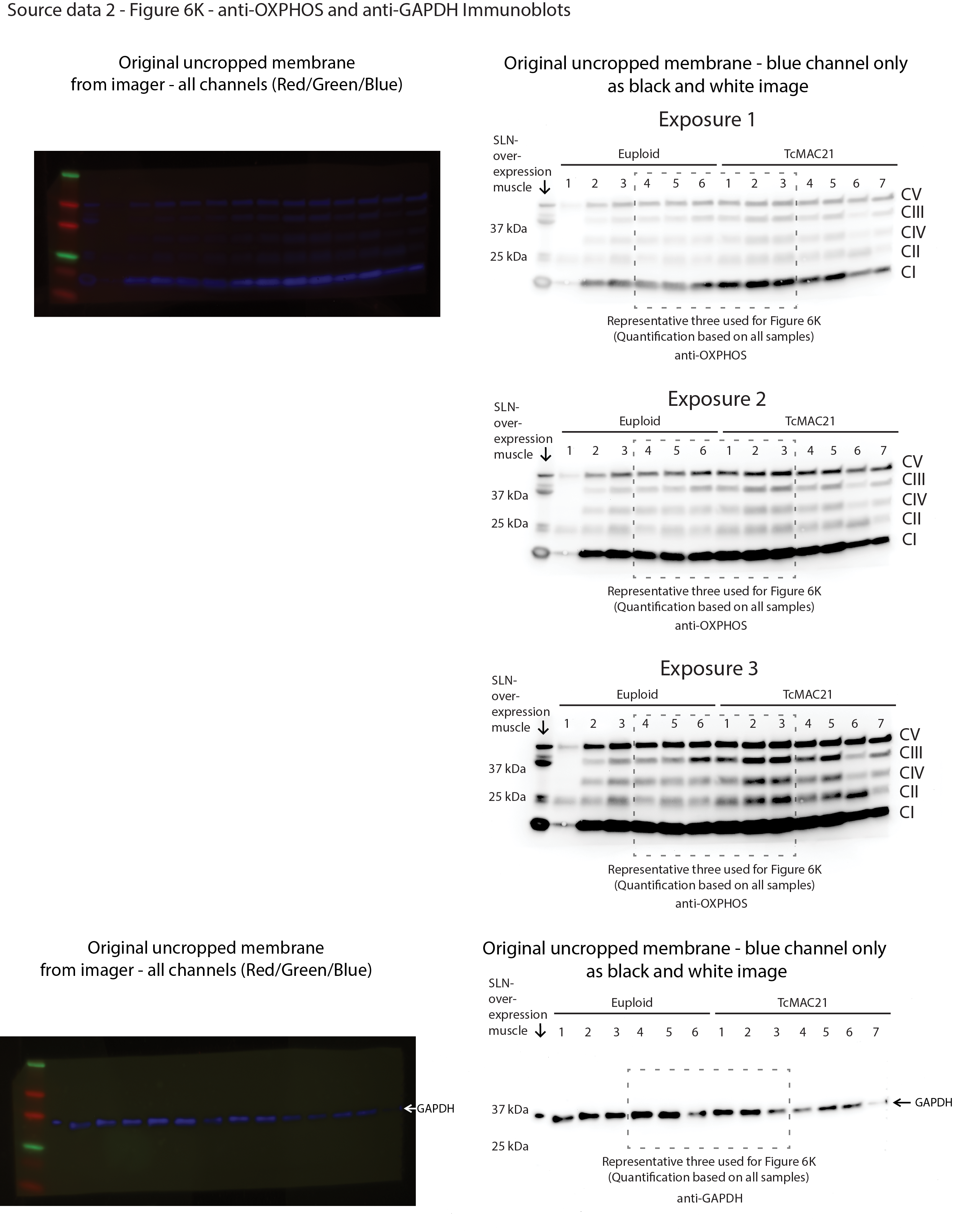

Supplement: Figure 6—source data 2. — Top right—Original uncropped membranes of different exposures showing black and white images of the blue channel only. OXHPHOS complex protein bands are labeled. The dotted outline represents where the membrane image was cropped for use in Figure 6. Bottom left—Original uncropped membrane from imager showing all channels (red/green/blue), with the GAPDH protein band labeled and appearing blue. Bottom right—Original uncropped membrane showing a black and white image of the blue channel only. GAPDH band is marked with an arrow. The dotted outline represents where the membrane image was cropped for use in Figure 6. [file elife-86023-fig6-data2.zip › Figure 6-source data 2/Figure 6-source data 2.tif]

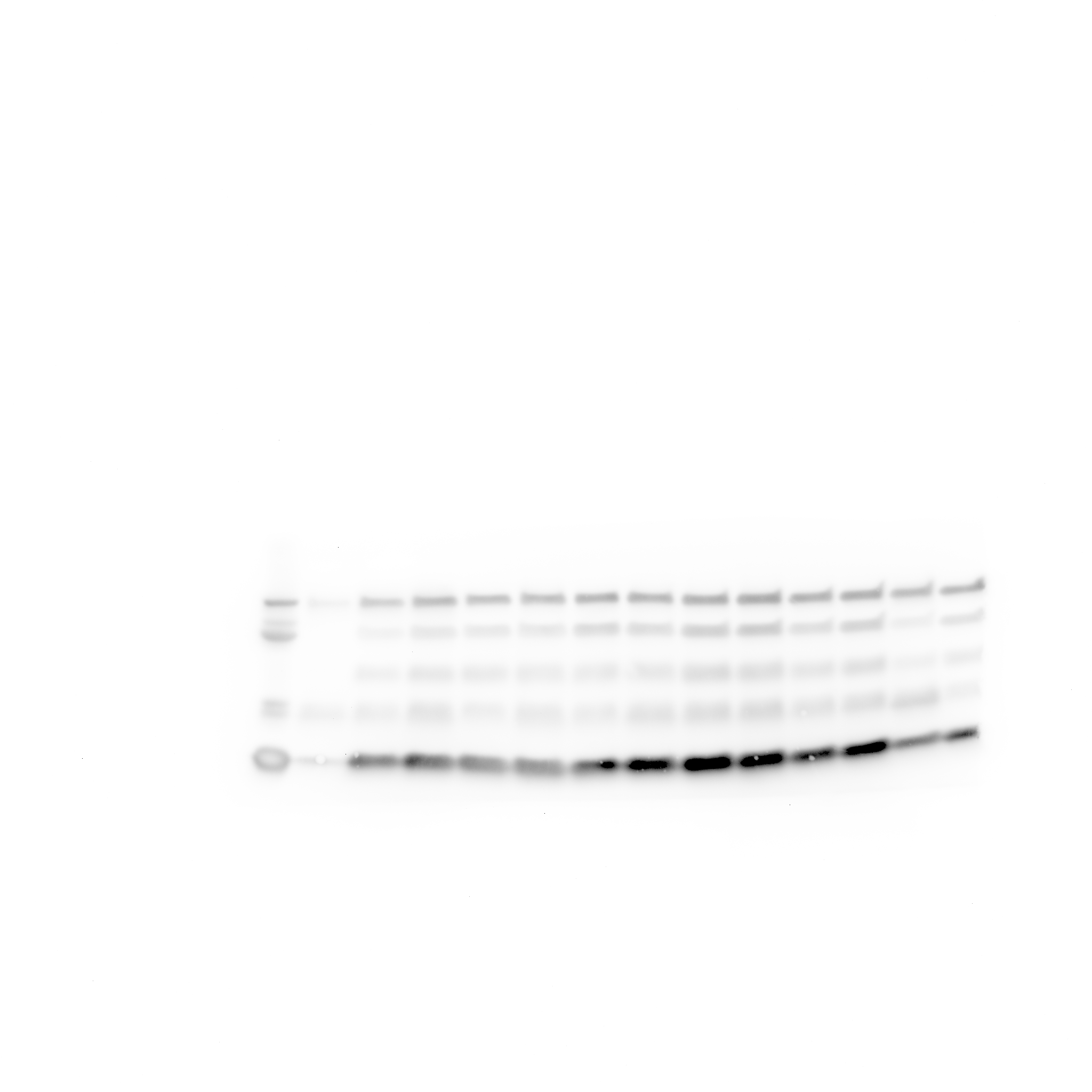

Supplement: Figure 6—source data 2. — Top right—Original uncropped membranes of different exposures showing black and white images of the blue channel only. OXHPHOS complex protein bands are labeled. The dotted outline represents where the membrane image was cropped for use in Figure 6. Bottom left—Original uncropped membrane from imager showing all channels (red/green/blue), with the GAPDH protein band labeled and appearing blue. Bottom right—Original uncropped membrane showing a black and white image of the blue channel only. GAPDH band is marked with an arrow. The dotted outline represents where the membrane image was cropped for use in Figure 6. [file elife-86023-fig6-data2.zip › Figure 6-source data 2/Figure 6-source data 2-Original uncropped membrane - bue channel only as black and white image-antiOXPHOS-exposure 1.tif]

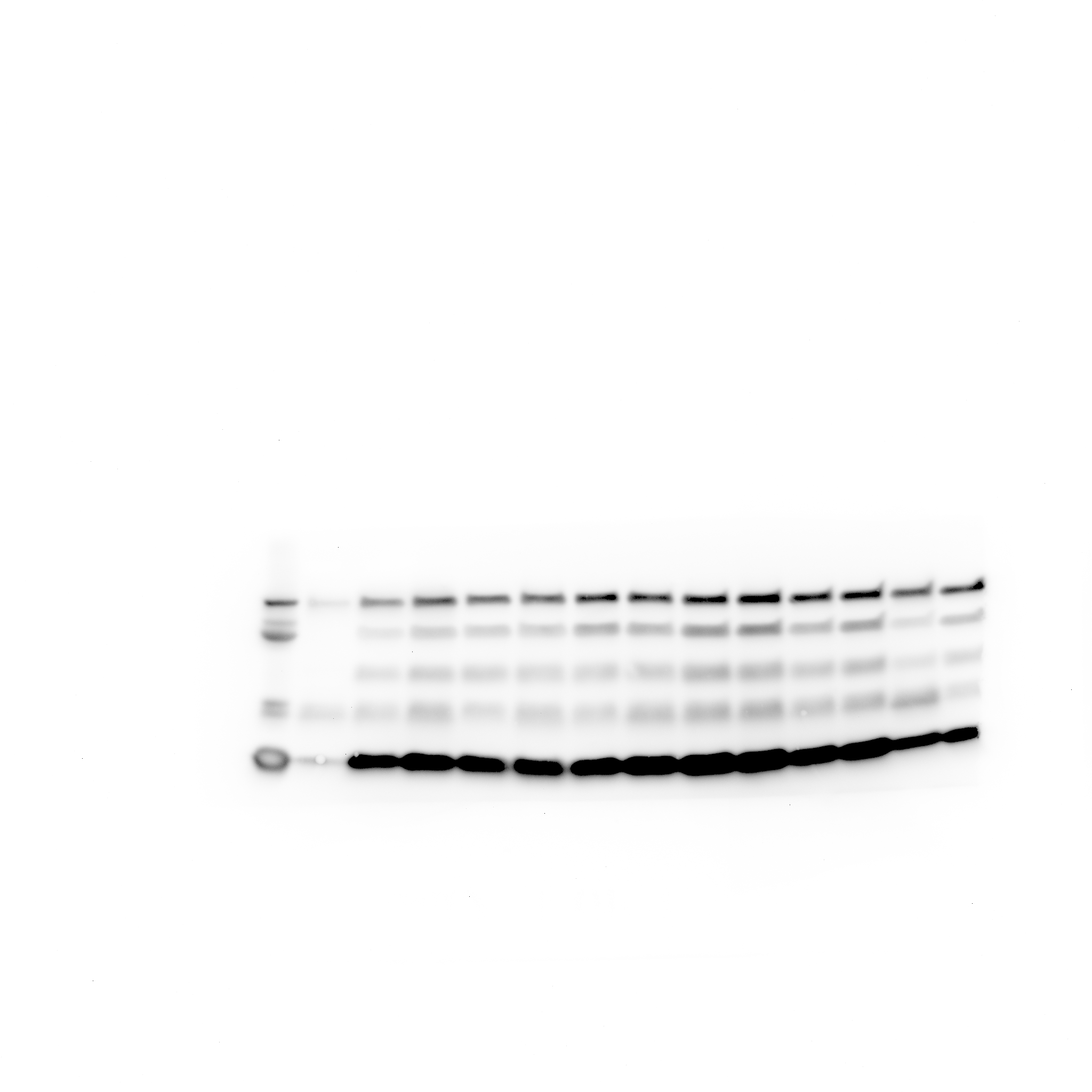

Supplement: Figure 6—source data 2. — Top right—Original uncropped membranes of different exposures showing black and white images of the blue channel only. OXHPHOS complex protein bands are labeled. The dotted outline represents where the membrane image was cropped for use in Figure 6. Bottom left—Original uncropped membrane from imager showing all channels (red/green/blue), with the GAPDH protein band labeled and appearing blue. Bottom right—Original uncropped membrane showing a black and white image of the blue channel only. GAPDH band is marked with an arrow. The dotted outline represents where the membrane image was cropped for use in Figure 6. [file elife-86023-fig6-data2.zip › Figure 6-source data 2/Figure 6-source data 2-Original uncropped membrane - bue channel only as black and white image-antiOXPHOS-exposure 3.tif]

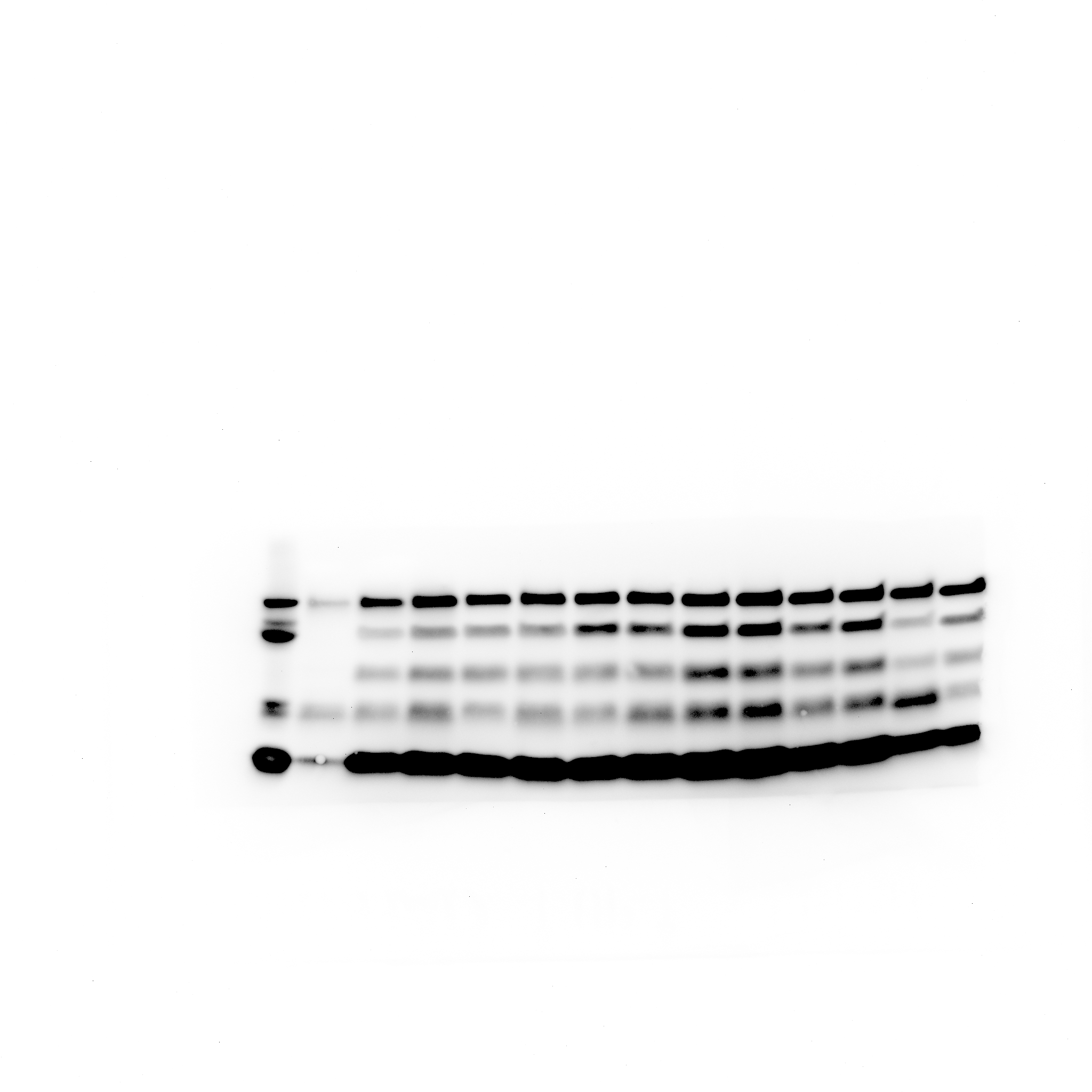

Supplement: Figure 6—source data 2. — Top right—Original uncropped membranes of different exposures showing black and white images of the blue channel only. OXHPHOS complex protein bands are labeled. The dotted outline represents where the membrane image was cropped for use in Figure 6. Bottom left—Original uncropped membrane from imager showing all channels (red/green/blue), with the GAPDH protein band labeled and appearing blue. Bottom right—Original uncropped membrane showing a black and white image of the blue channel only. GAPDH band is marked with an arrow. The dotted outline represents where the membrane image was cropped for use in Figure 6. [file elife-86023-fig6-data2.zip › Figure 6-source data 2/Figure 6-source data 2-Original uncropped membrane - bue channel only as black and white image-antiOXPHOS-exposure 2.tif]

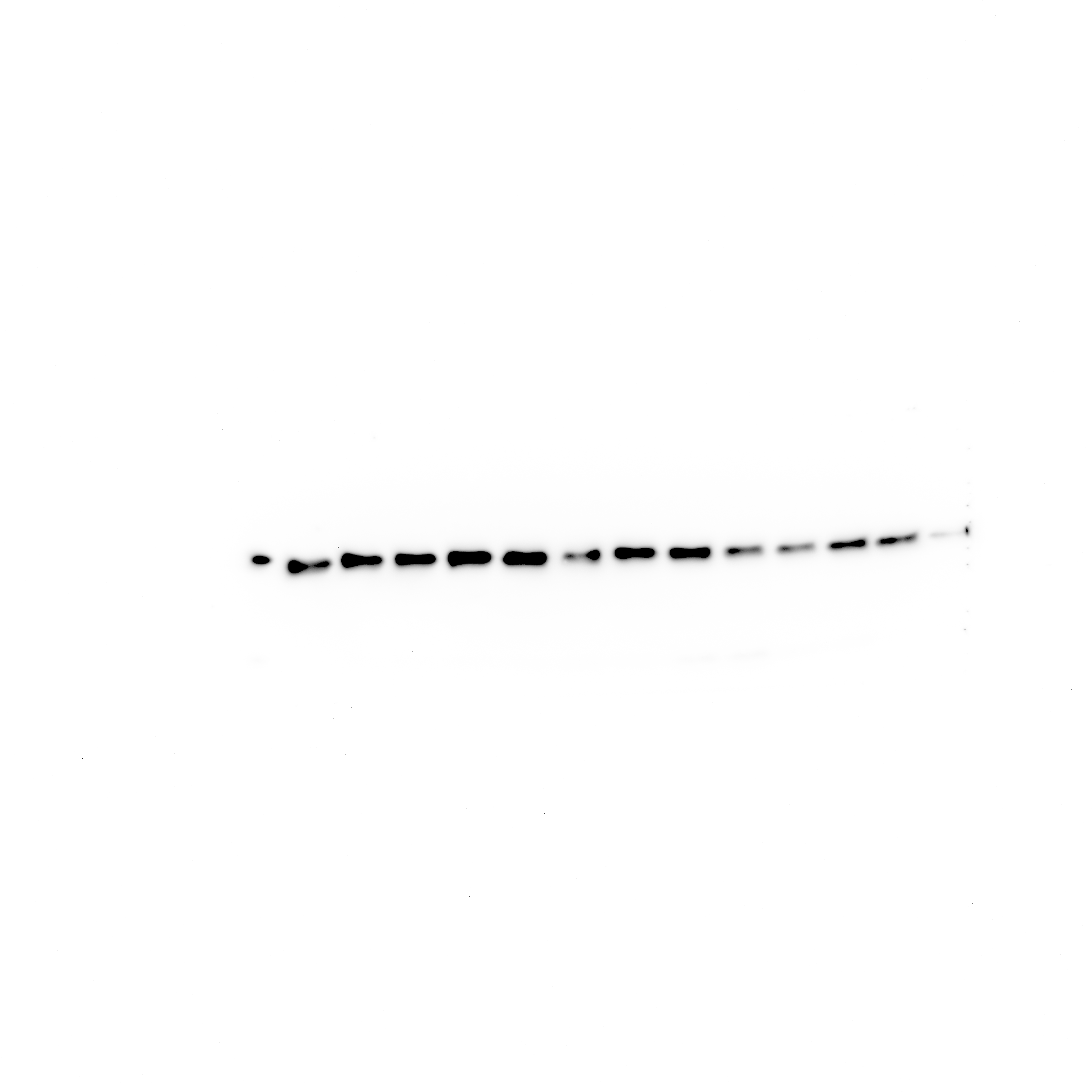

Supplement: Figure 6—source data 2. — Top right—Original uncropped membranes of different exposures showing black and white images of the blue channel only. OXHPHOS complex protein bands are labeled. The dotted outline represents where the membrane image was cropped for use in Figure 6. Bottom left—Original uncropped membrane from imager showing all channels (red/green/blue), with the GAPDH protein band labeled and appearing blue. Bottom right—Original uncropped membrane showing a black and white image of the blue channel only. GAPDH band is marked with an arrow. The dotted outline represents where the membrane image was cropped for use in Figure 6. [file elife-86023-fig6-data2.zip › Figure 6-source data 2/Figure 6-source data 2-Original uncropped membrane - bue channel only as black and white image-antiGAPDH.tif]

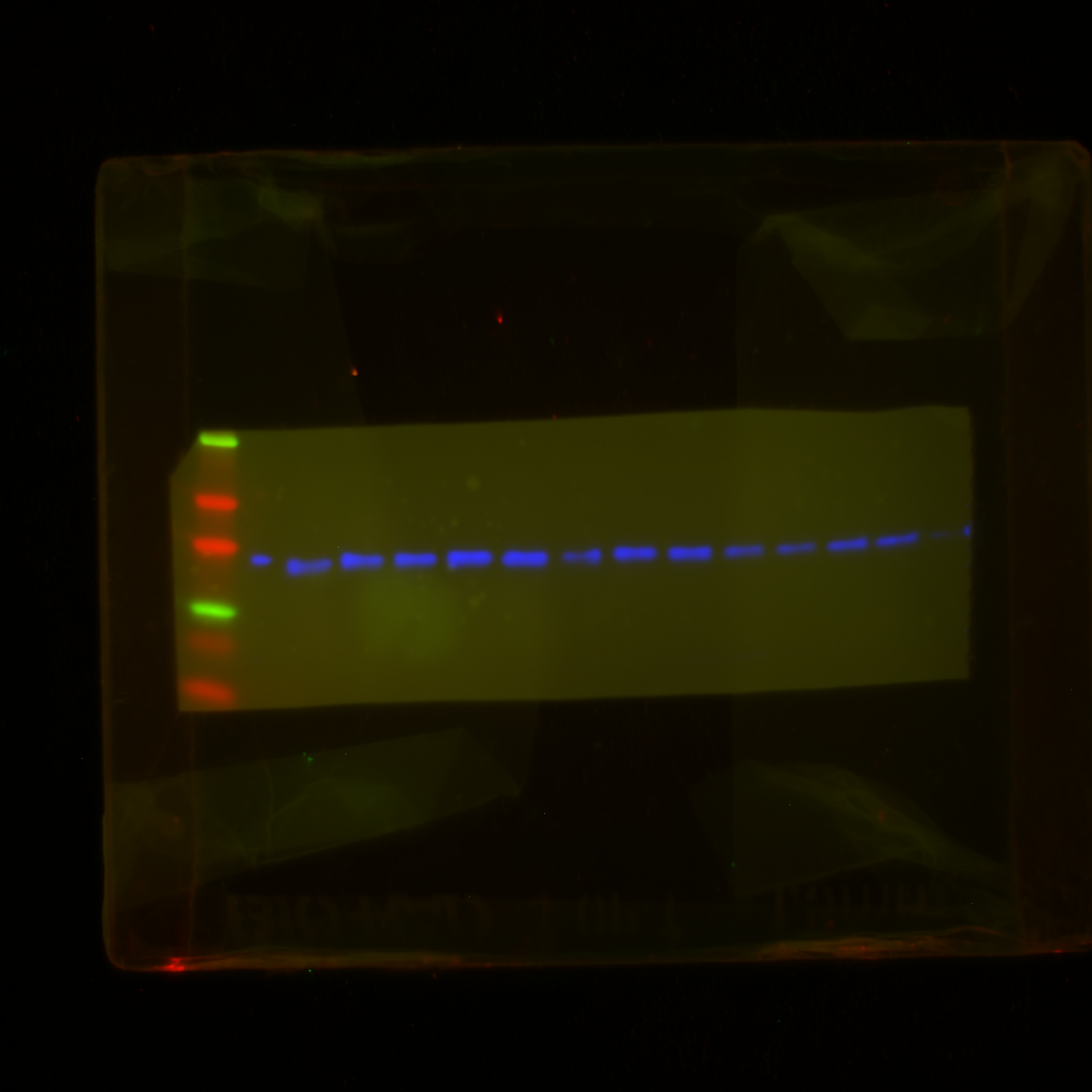

Supplement: Figure 6—source data 2. — Top right—Original uncropped membranes of different exposures showing black and white images of the blue channel only. OXHPHOS complex protein bands are labeled. The dotted outline represents where the membrane image was cropped for use in Figure 6. Bottom left—Original uncropped membrane from imager showing all channels (red/green/blue), with the GAPDH protein band labeled and appearing blue. Bottom right—Original uncropped membrane showing a black and white image of the blue channel only. GAPDH band is marked with an arrow. The dotted outline represents where the membrane image was cropped for use in Figure 6. [file elife-86023-fig6-data2.zip › Figure 6-source data 2/Figure 6-source data 2-Original uncropped membrane from imager - all channels (Red:Green:Blue)-antiGAPDH.tif]

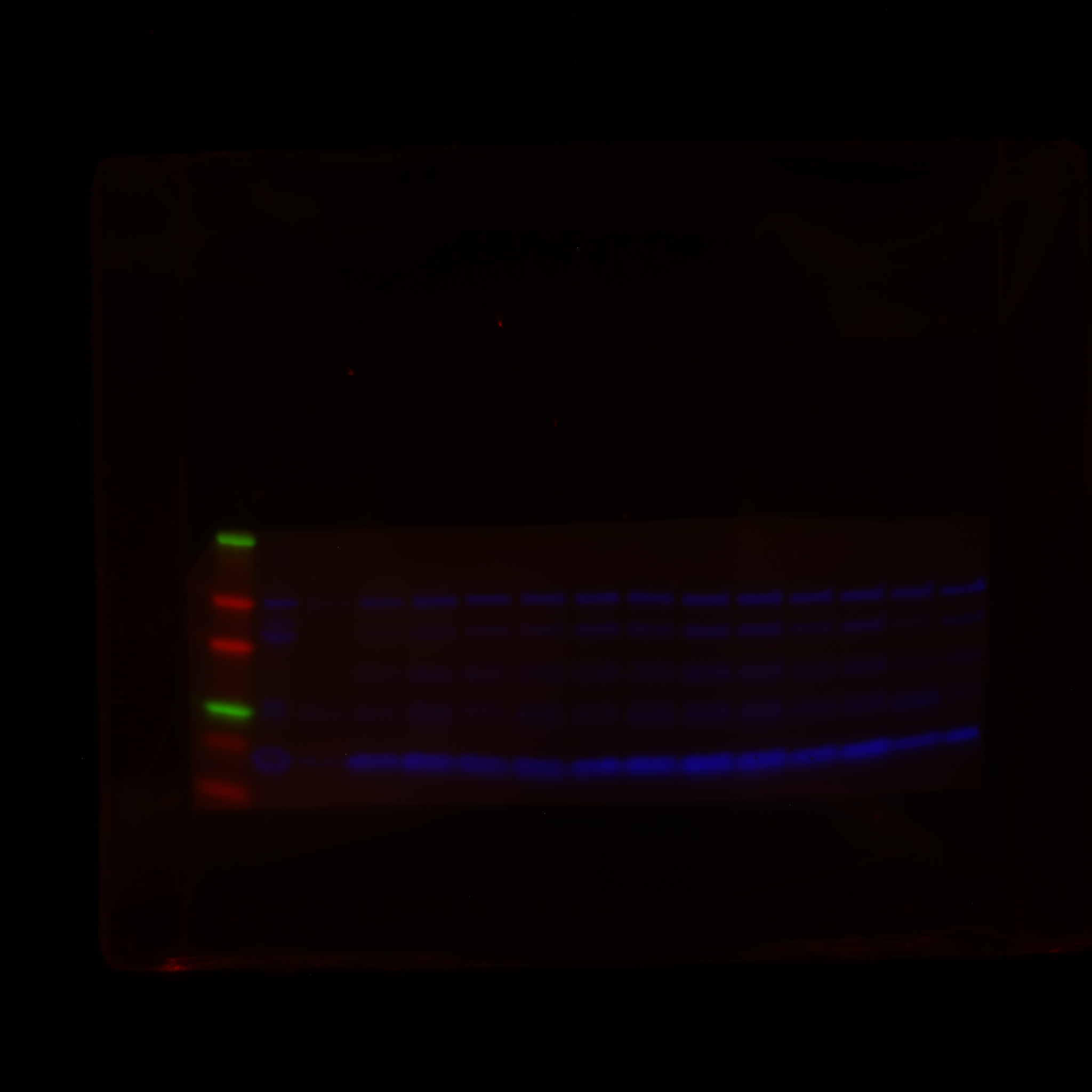

Supplement: Figure 6—source data 2. — Top right—Original uncropped membranes of different exposures showing black and white images of the blue channel only. OXHPHOS complex protein bands are labeled. The dotted outline represents where the membrane image was cropped for use in Figure 6. Bottom left—Original uncropped membrane from imager showing all channels (red/green/blue), with the GAPDH protein band labeled and appearing blue. Bottom right—Original uncropped membrane showing a black and white image of the blue channel only. GAPDH band is marked with an arrow. The dotted outline represents where the membrane image was cropped for use in Figure 6. [file elife-86023-fig6-data2.zip › Figure 6-source data 2/Figure 6-source data 2-Original uncropped membrane from imager - all channels (Red:Green:Blue)-antiOXPHOS.tif]
